# Running trends in Switzerland from 1999 to 2019: An exploratory observational study

**DOI:** 10.1371/journal.pone.0311268

**Published:** 2025-01-16

**Authors:** Anja Witthöft, Thimo Marcin, Mabliny Thuany, Volker Scheer, Pantelis T. Nikolaidis, Matthias Wilhelm, Katja Weiss, Thomas Rosemann, Beat Knechtle

**Affiliations:** 1 Centre for Rehabilitation & Sports Medicine, Inselspital, University Hospital of Bern, University of Bern, Bern, Switzerland; 2 Berner Reha Zentrum, Rehabilitation & Sports Medicine, Insel Group, Bern University Hospital, Bern, Switzerland; 3 Department of Physical Education, State University of Para, Pará, Brazil; 4 Ultra Sports Science Foundation, Pierre-Benite, France; 5 School of Health and Caring Sciences, University of West Attica, Athens, Greece; 6 Institute of Primary Care, University of Zurich, Zurich, Switzerland; 7 Medbase St. Gallen Am Vadianplatz, St. Gallen, Switzerland; Universidade Federal de Minas Gerais, BRAZIL

## Abstract

**Background:**

Several single race events (5 km, 10 km, half-marathon, marathon, ultra-marathon) in different countries and different years have been analyzed in multiple studies, representing the rising interest in endurance-based activity and thus physical health. With focus on participation numbers, performance or sex difference, many single study results were obtained. The running trends in a whole country covering a longer period of time and several race distances are missing so far.

**Objectives:**

The aim of this study is therefore to examine 5 km, 10 km, half-marathon, marathon and ultra-marathon races by age, sex, participation numbers and performance during two decades (1999–2019) for one country (Switzerland).

**Methods:**

In this exploratory observational study, we analyzed 1,172,836 finishers (370,517 women and 802,319 men) competing between 1999 and 2019 in 5 different race distances in Switzerland. We used publicly available data about the athletes and examined total finishing numbers, sex, age and performances (measured in m/s) via descriptive analyses and linear mixed models. Do-not-finishers were excluded.

**Results:**

The most frequented race was the half-marathon (33.1% of finishers), the less frequented was the ultra-marathon distance (8.5% of finishers). In most recent years, only the number of finishers in ultra-marathon, especially in trail runs increased. In total, there were more male finishers (68.4%) than female finishers (31.6%) and only in 5 km races, more women finished than men (55.3%). Men were faster than women and both sexes were running slower in all race distances across years. Athletes in 10 km races had the best performance within the five analyzed race distances. Median age increased with longer race distance and decreased in ultra-marathon in recent years.

**Conclusion:**

In summary, finishing numbers especially in ultra-marathons increased with a focus on trail runs, female and male athletes had a declining performance across years in all race distances and men ran faster than women. Median age increased with longer race distance leading to more aged endurance-trained athletes. A downtrend in median age is found only in ultra-marathon in recent years. The results are important for athletes, race directors and coaches with regard to training schedules and the trend towards long distance races as well as for the medical attendance especially of older athletes, being more and more interested in endurance running.

## Introduction

Running events of different distances gained in popularity for both elite and recreational runners all over the world [1]. Taking into account the running-related characteristics (such as being easy to be performed; low cost when compared to other sports; accessible for most people, due to the possibility to manage the time and the place of training), running mass events have become a social phenomenon [2]. The positive effects of regular participation in endurance sports are well known: they lead from improved cardio-vascular fitness and weight management to the reduced risk of different chronic diseases (e.g., hypertension or diabetes) and the influence to mental well-being [3–5]. With the ageing society, the number of elderly people participating in endurance events has increased and leads to a deceleration of the human aging process [6,7].

When it comes to the different race distances, especially shorter running distances such as 5 km and 10 km races are counted among the most popular events with millions of participants [8,9]. Across years, they have attracted more and more athletes leading to a significant increase in the number of participants [8,9] especially in middle age group runners (20–49 years) and older runners (> 50 years) [10]. Younger runners were the fastest [10–12], while pace in 5 km races decreased over the years [10] and increased in 10 km races [11]. In both race distances, there is a focus on female participants [10,11], with even a higher finishing rate of women compared to men in 10 km races [9,11]. Men had better times than women in both short race distances [9,10].

In longer races such as the half-marathon, the number of finishers has seen an increase in the USA and in Switzerland until 2014 [8,12], but a decrease afterwards. Compared to a marathon, the half-marathon races in Switzerland showed clearly higher participation numbers [12,13]. However, in U.S.- marathon running, there has been an increase in the number of finishers between 1976 and 2014 [6,14,15]. More men than women finished successfully the longer distance races [9], and there were less female marathoners compared to half-marathoners [12]. Regarding age, half-marathoners in Switzerland were younger [12], whereas in the USA, marathoners were younger [16]. Women in half-marathon were older than their male counterparts [17], but in marathon races, they participated in younger age groups than men [18]. Again, the youngest half-marathoners were the fastest runners [19] and the oldest runners in marathon remained the slowest [9]. With respect to performance, half-marathoners were running slower than marathoners [12]. There are still contradictions between female and male performance, being unclear if men or women were running faster in half-marathon [12,20,21], but it is known that female runners scored their best race time in marathon five years earlier in life than men [18]. We have data for half-marathon trends for Switzerland until 2014 and for the USA until 2016, but more recent data from the last years are missing, especially for sex and age group trends, as well as data covering several race distances in a country.

When addressing ultra-marathons, which are defined as any running distance or time-limited running events counting for more than the traditional 42.195 km of a marathon or exceeding 6 hours of duration [22,23], they have seen as well an exponential increase in the number of finishers and the number of ultra-marathon events [24,25], which take place on road or trails. A trail run is defined as a foot race in a natural environment (mountains, deserts, forests etc.) over a variety of different terrains (dirt road, forest trail, single track, beach sand etc.) with minimal paved or asphalt roads, not exceeding 25% of the total race course, with no limit to running distance [23].

Ultra-marathons still see 80% of male finishers [24,26,27], even if women participate increasingly in running events. Regarding performance trends, there is a constant improvement [28], with a faster performance in men than in women [29] as well as in older athletes (>70 years). Older athletes also showed a smaller performance gap between men and women [30]. Only the youngest women in ultra-marathon (20–29 years) even had similar performance levels than men [31]. The performance gap between the sexes varies by race distance [29] and a higher age of peak ultra-marathon performance comes along with a longer race duration [32].

In summary, many studies have been analyzing single large events in different countries with a limited number of participants [33–36]. At the European level, available data estimates that approximately 45–55 million people are involved in running [37]. However, no study has so far analyzed the running trends for several running distances and endurance events over a longer period of time for a single country. Switzerland, as showing one of the highest rates of sports practice in population throughout Europe, is well suited for this analysis.

Therefore, the aim of the present retrospective study was to analyze the participation and performance trends of age group athletes competing in 5 km, 10 km, half-marathon, marathon and ultra-marathon races held between 1999 and 2019 in a whole country, Switzerland. We evaluated the ratio of male and female finishers as well as their age distribution and differences in performance.

## Methods

### Ethical approval

All procedures used in the study were approved by the Institutional Review Board of Kanton St. Gallen, Switzerland with a waiver of the requirement for informed consent of the participants given the fact that the study involved the analysis of publicly available data (EKSG 01/06/2010). Names of the participants were not used for the following analyses.

### Study design, data sampling and data analysis

In this exploratory observational study, we collected publicly and online available data from 5 and 10 km races, half-marathon, marathon and ultra-marathon held in Switzerland between 1999 and 2019 from different sources such as „swiss-running”[38], „runme”[39], „datasport”[40] and the official „DUV-Homepage”[41]. The data was collected on 14/15/16 January 2021 and 31 July 2021. Due to the Covid-19 pandemic, we decided to stop collecting data after 2019 as most of the races were cancelled in the following years or only took place under special conditions, which would have led to inconsistent data. Moreover, we collected only data with clear indication of the running distance. Ultra-marathons were classified into distance-limited (longer than 42.195 km) and time-limited (6 hours and longer) events. We included both road races and trail runs. Respecting a given error of measurement, we included distances deviating +/- 1 km from the distances mentioned above. There are no other eligibility criteria.

The athlete data such as name, surname, year of birth, sex, race distance and race time were downloaded and converted into an Excel file. Names were not used for the analyses. Age was derived by subtracting the year of birth from the year in which the race took place. Race times were converted to running speed (km/h and m/s) using the race distances (km) and the race time (h:min:s, ms). Athletes were classified into 5-year age groups, i.e., <15, 15–19, 20–24, … ≥80 years.

A total of 1,172,836 finishers were considered in this study as well as a total number of 1109 events and 166 races. Athletes with incomplete data (e.g. missing age), seemingly incorrect data (such as 1 or 99 years old, as often inserted if age is unknown) or did-not-finishers (DNF) were not included in the analysis. In terms of numbers, we excluded 15,442 DNF and 1,657 runners with missing data. The DNF were not considered in the study, as the number of DNF is not complete due to a lack of online available data.

When the terms “athletes” or “runners” are used in the text, we also mean “finishers”. Moreover, it should be noted that several finishers participated in several races or ran the same race in different years. Due to omitting the names for the analyses, they are therefore counted several times. This also means that we were not able to count persons, but only the records of athletes finishing a race.

### Statistical analysis

All statistics were performed with R (Version 3.5.1, R Core Team, 2017). Trends of finishing rate over the years were descriptively analyzed using counts of finishers. Trends of finishing rate, i.e., number of finishers by calendar year are shown for each race distance overall and women and men separately. Age distribution (median and quartiles) is graphically shown by race distance and calendar year. Performance trends were measured in m/s. For trend analyses of running speed (m/s) over the years, the top-3, top-10 and top-100 of each age-group and sex within every single competition were tagged. We performed linear mixed models for running speed (m/s for each running distance and finisher level (top-3, top-10, top-100 and overall)) separately with following independent variables: year*sex interaction, age as fixed factor and competition as random intercepts. For better illustration, the predicted pace (m/s) with its 95% confidence intervals was derived from the linear mixed models and graphically illustrated using plot_model function from R package sjPlot.

To obtain an estimate for the linear change over the years, we performed additional linear mixed models separately for each finisher level and sex with following year as continuous independent variable adjusted for competition as random intercept. The estimates with 95% confidence intervals for the independent variable year were sought from the models to illustrate the change of pace over the years for the different sex and finisher levels.

## Results

The 1,172,836 analyzed finishers in 224 different race events repeating every year were divided into the different race distances as seen in Table 1. The most frequented race across years in terms of finishers was the half-marathon, the less frequented was the ultra-marathon. Concerning the sex ratio, 68.4% were male finishers and 31.6% were female finishers. Only in 5 km races, more women (55,3%) participated compared to men (44.7%).

**Table 1:** Number of finishers by sex and race distance as well as number of different race events (repeating each year).

	5 km race	10 km race	Half-marathon	Marathon	Ultra-marathon	Total
**Total finishers**	114,363	338,537	387,950	232,034	99,952	1,172,836
**Total number of finishers (%)**	9.8	28.9	33.1	19.7	8.5	100
**Male finishers**	51,131	209,816	270,077	188,237	83,058	802,319
**Female finishers**	63,232	128,721	117,873	43,797	16,894	370,517
**Male finishers/ distance (%)**	44.7	62.0	69.6	81.1	83.1	68.4
**Female finishers/ distance (%)**	55.3	38.0	30.4	18.9	16.9	31.6
**Men-to-women-ratio (MWR)**	0.8	1.6	2.3	4.3	4.9	2.2
**Number of different race events**	32	49	30	15	98	224

### 5 km races

**Fig 1:**
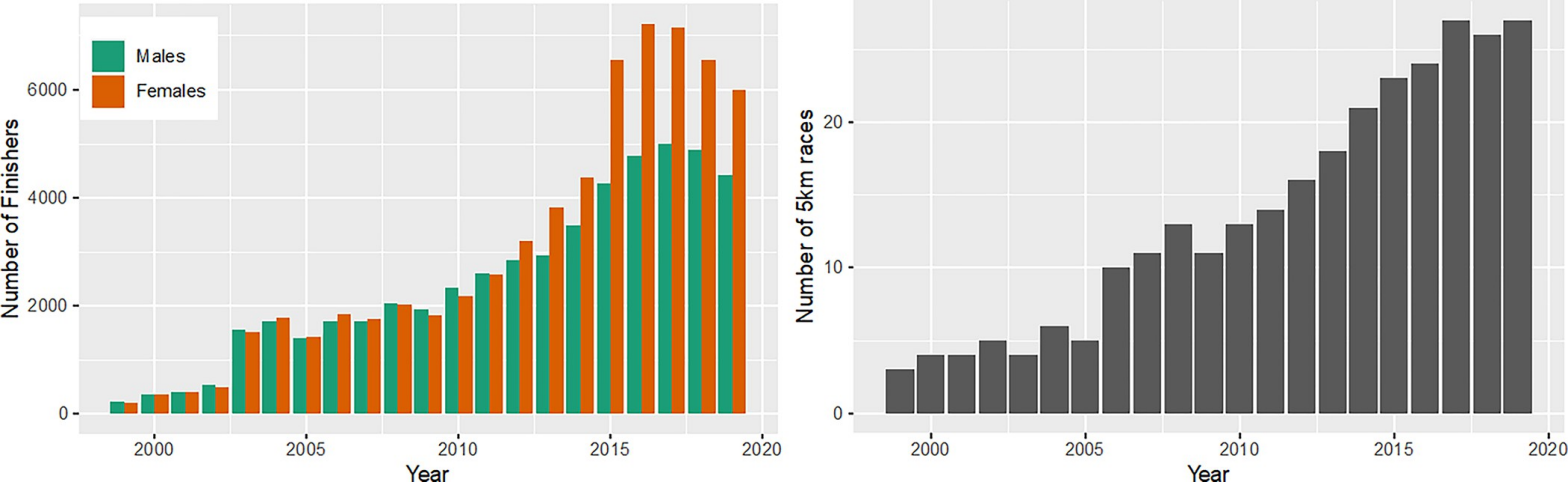
Absolute numbers of female and male finishers in 5 km races and number of 5 km races. Female and male finishers in 5 km races over calendar years (panel left); number of 5 km races over calendar years (panel right).

The number of finishers in 5 km races increased from 1999 to 2017 and showed a slight decrease in 2018 and 2019 (Fig 1, panel left). While the men-to-women-ratio (MWR) was nearly similar from 1999 to 2011 (mean 1.0, SD 0.1), more women than men finished in 5 km races from 2012 on (Fig 1, panel left). The highest female finishing rate was observed in 2016 with 7,149 runners, whereas the strongest male finishing rate was in 2017 with 4,494 male runners. A decrease in the numbers of finishers of both sexes was again seen in recent years (2018 and 2019), while women were still the majority. The number of 5 km races increased significantly (p<0.001) across the past 20 years, with a minimum of 3 races in 1999 and a maximum of 27 races in 2017 and 2019 (Fig 1, panel right).

### 10 km races

**Fig 2:**
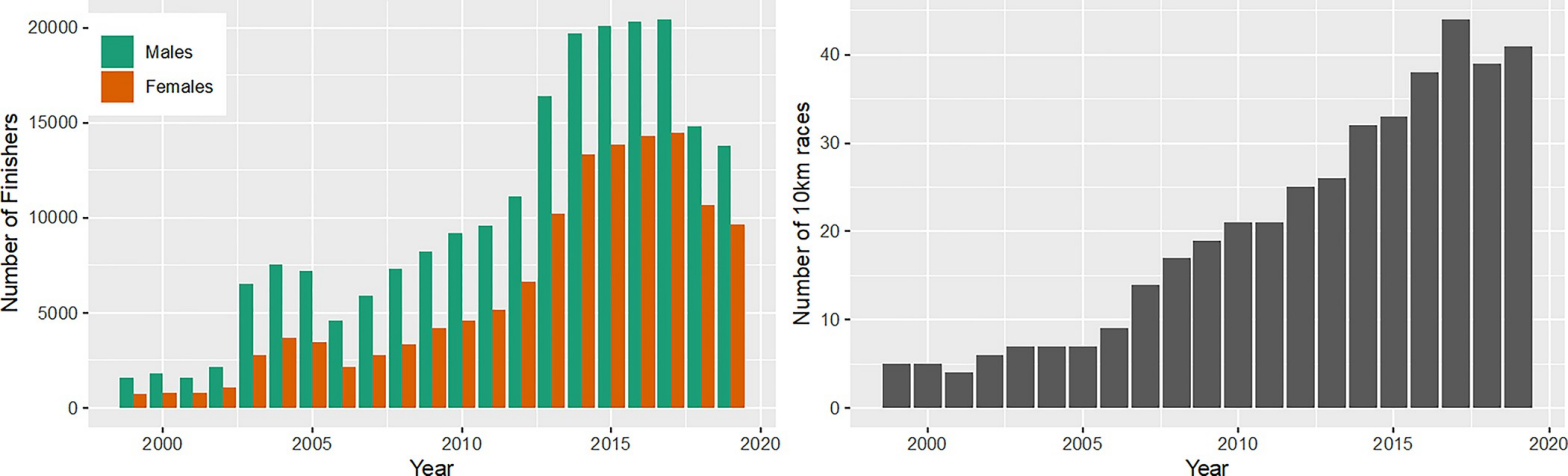
Absolute numbers of female and male finishers in 10 km races and number of 10 km races. Female and male finishers in 10 km races over calendar years (panel left); number of 10 km races over calendar years (panel right).

The number of finishers in 10 km races increased from 1999 to 2017 with a decrease in 2018 and 2019 (Fig 2, panel left). Concerning the absolute numbers of female and male finishers across the years, more men than women finished in 10 km races with an increase in both sexes until 2017. The highest finishing rate for both sexes was observed in 2017 with 20,426 male runners and 14,489 female runners. In recent years (2018 and 2019), the number of finishers has decreased for both sexes (Fig 2, panel left), while men were still the majority. The number of 10 km races increased significantly up to 44 races in 2017 (Fig 2, panel right).

### Half-marathon races

**Fig 3:**
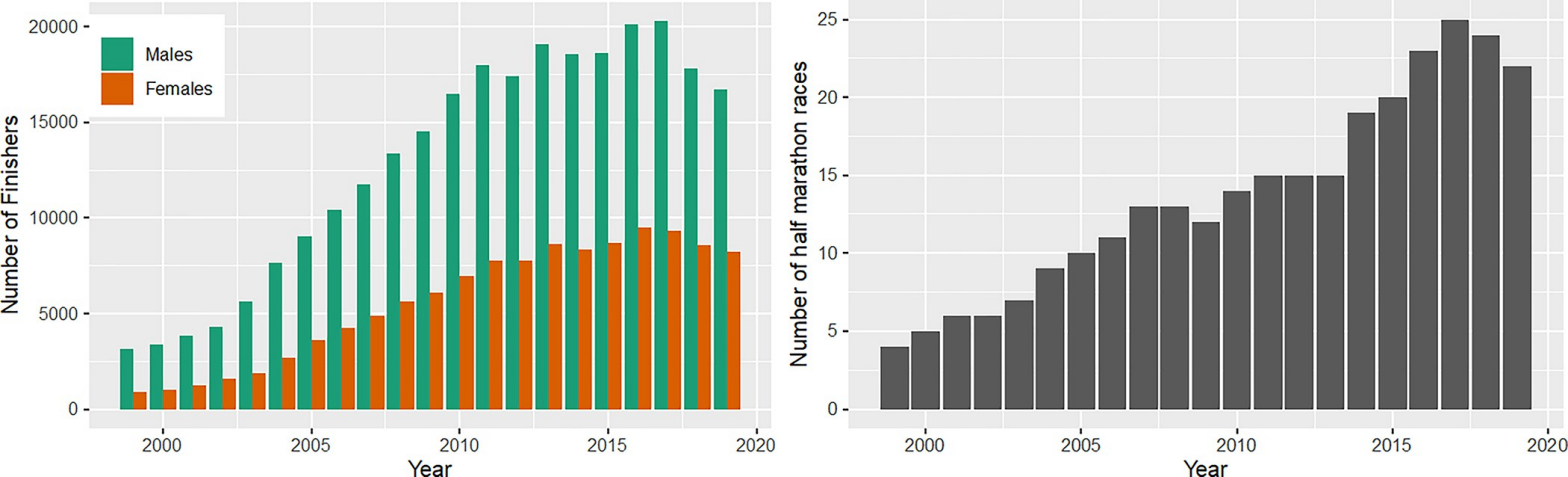
Absolute numbers of female and male finishers in half-marathon races and number of half-marathon races. Female and male finishers in half-marathon races over calendar years (panel left); number of half-marathon races over calendar years (panel right).

The number of finishers in half-marathons increased from 1999 to 2017 with a flattening of the curve after 2012. A decrease in the number of finishers is seen in 2018 and 2019 (Fig 3, panel left). Across the past 20 years, there were constantly more men than women finishing in half-marathon (Fig 3, panel left). The maximum of male finishers was 20,270 in 2017, while the maximum of female finishers was 9,530 runners in 2016. A decrease in both sexes was again seen in recent years (2018 and 2019). The number of half-marathon events increased from 1999 to 2017 from 4 to 25 races (Fig 3, panel right).

### Marathon races

**Fig 4:**
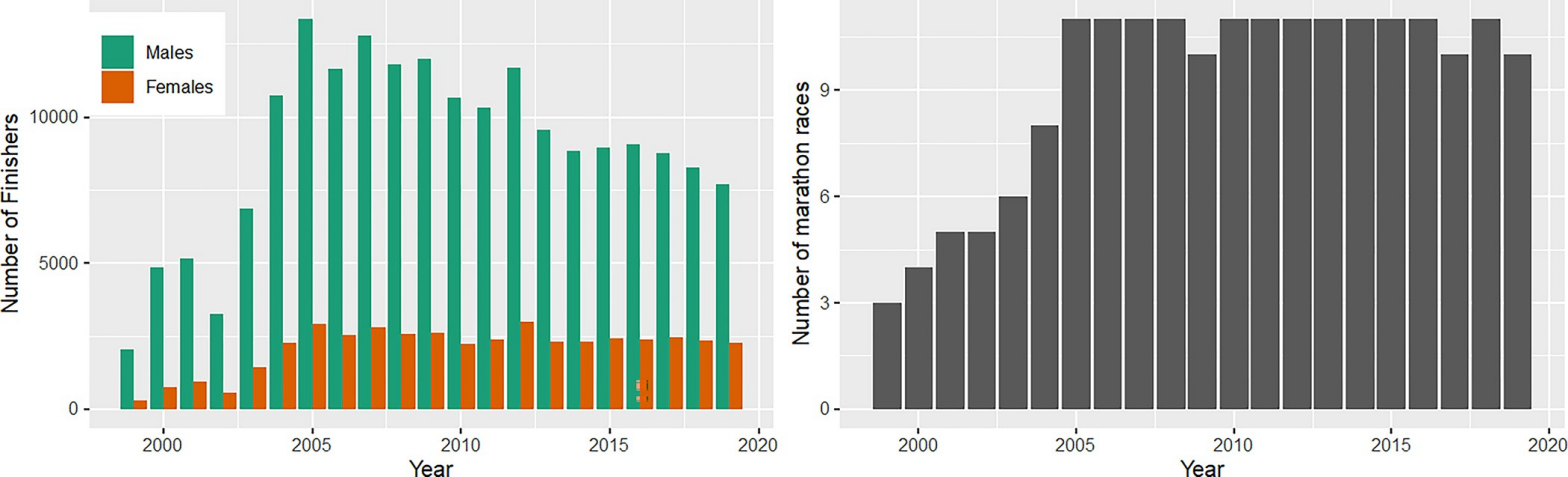
Absolute numbers of female and male finishers in marathon races and number of marathon races. Female and male finishers in marathon races over calendar years (panel left); number of marathon races over calendar years (panel right).

The number of finishers in marathons increased from 1999 to 2005 and showed a progressive decrease thereafter (Fig 4, panel left). Across the past 20 years, there were constantly more men than women finishing in marathon with a maximum of 13,351 male finishers in 2005 and a decrease thereafter. The numbers of female finishers increased until 2005 up to 2,909 athletes and remained stable afterwards (Fig 4, panel left). Since 2005, the number of marathon races remained stable with 10–11 races per year. A minimum of 3 marathon races was shown in 1999 (Fig 4, panel right).

### Ultra-marathon races

**Fig 5:**
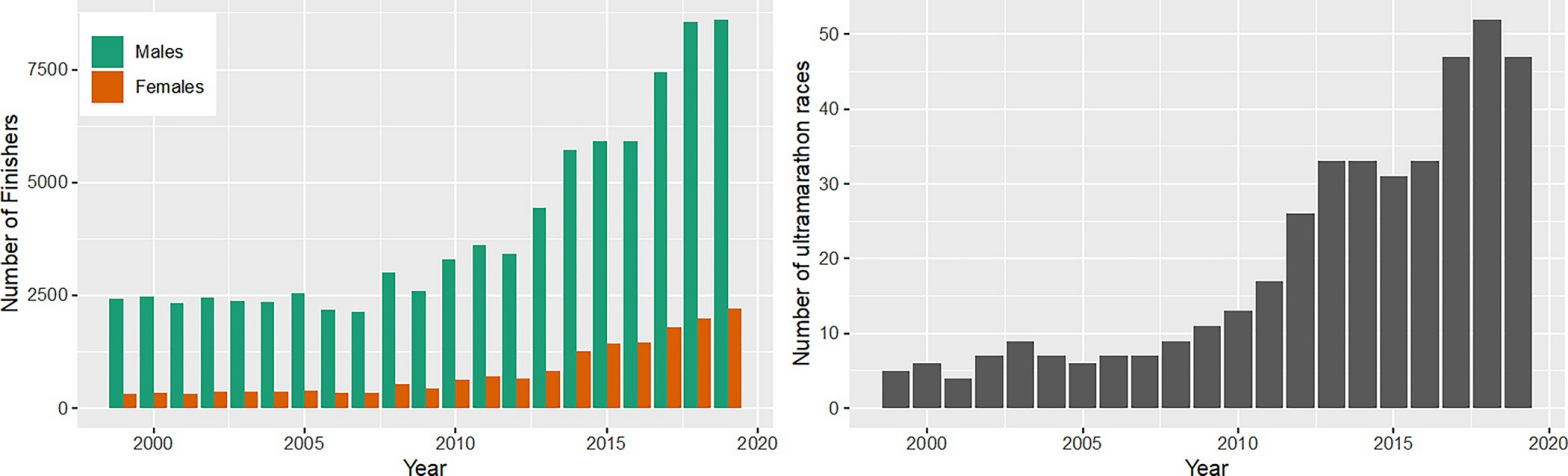
Absolute numbers of female and male finishers in ultra-marathon races and number of ultra-marathon races. Female and male finishers in ultra-marathon races over calendar years (panel left) and number of ultra-marathon races over calendar years (panel right).

**Fig 6:**
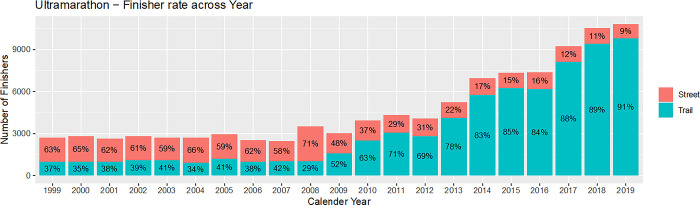
Ultra-marathon: Finisher rate in street races and trails runs over calendar years.

The number of finishers in ultra-marathons remained stable from 1999 to 2009 and showed an increase from 2010 to 2019 (Fig 5, panel left). Across the past 20 years, there was an increase in finishers for both sexes, even if there were constantly more men than women finishing in ultra-marathons. The number of finishers in ultramarathon increased continuously in both sexes with up to 2,213 female and 8,611 male finishers in 2019 (Fig 5, panel left). The number of ultra-marathons increased largely after 2008 from 9 to 33 races in 2016 and 52 races in 2018 (Fig 5, panel right). There was a large increase of finishers in trail runs, while the numbers of finishers in ultra-marathons on street decreased from 2,519 finishers in 2008 to 1,020 finishers in 2019 (Fig 6).

### Median age by race distance and calendar year

**Fig 7:**
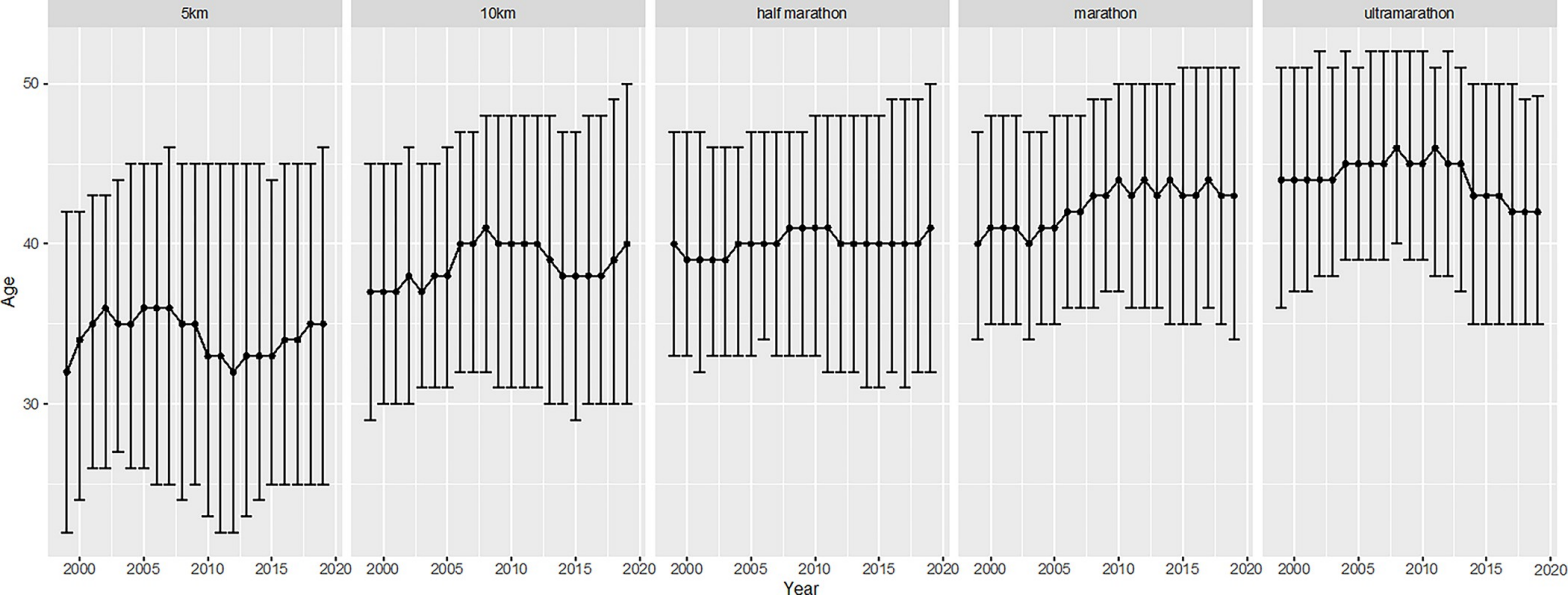
Median (Quartiles) age of finishers by year and race distance. Error bars indicate the upper and lower quartiles of age corresponding to one year per race distance with the median age of finishers as solid line over the years.

Fig 7 shows the age distribution (median, quartiles) by race distance and calendar year. The average age from 1999–2019 was lowest in 5 km races (34 years) and was found to be higher with increasing distance (44 years in ultra-marathon). Across the years, median age in finishers increased in 5 km races, 10 km races and marathon, whereas it decreased in ultra-marathon after 2012. In half-marathon, the median age of finishers remained relatively stable with 40 years over the years.

### Number of finishers by race distance and calendar year

**Fig 8:**
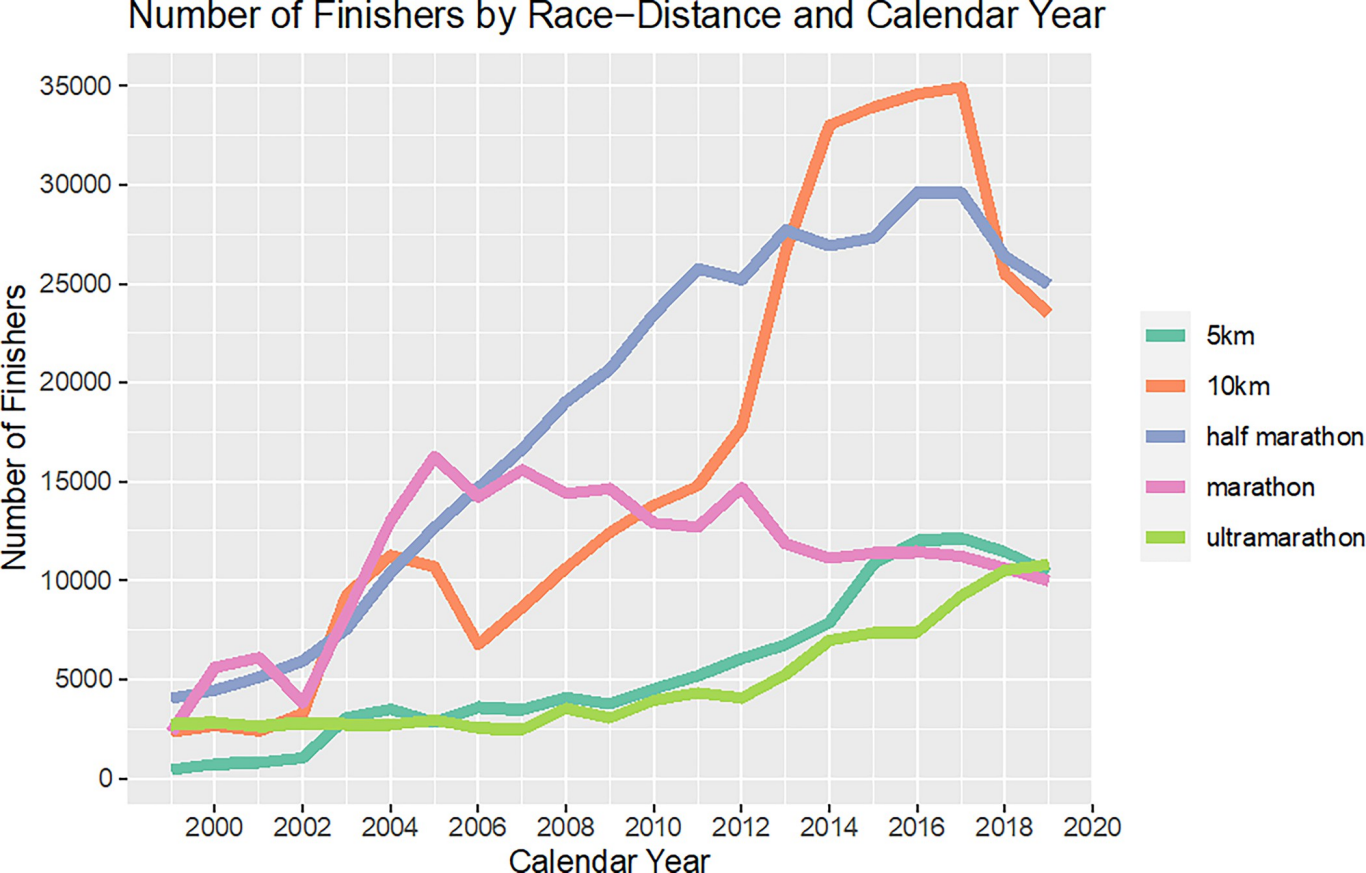
Overview: Number of finishers by race distance and calendar year.

An increase in the number of finishers by race distance and calendar year is seen in 5 km races, 10 km races and half-marathons across the years, with a decrease in recent years (Fig 8). In ultra-marathons, there was a constant increase in finishers, even in recent years. The number of finishers in marathon was constantly decreasing after a peak in 2005. In total, 10 km races and half-marathons showed the highest numbers of finishers per year with a mean (SD) of 16,134 (11,339) and 18,483 (9,199) respectively. The lowest number of finishers per year was observed in 5 km races and in ultra-marathon, with a mean (SD) of 5,454 (3,895) and 4,800 (2,762) respectively.

### Running speed by race distance, year, sex and rank

**Fig 9:**
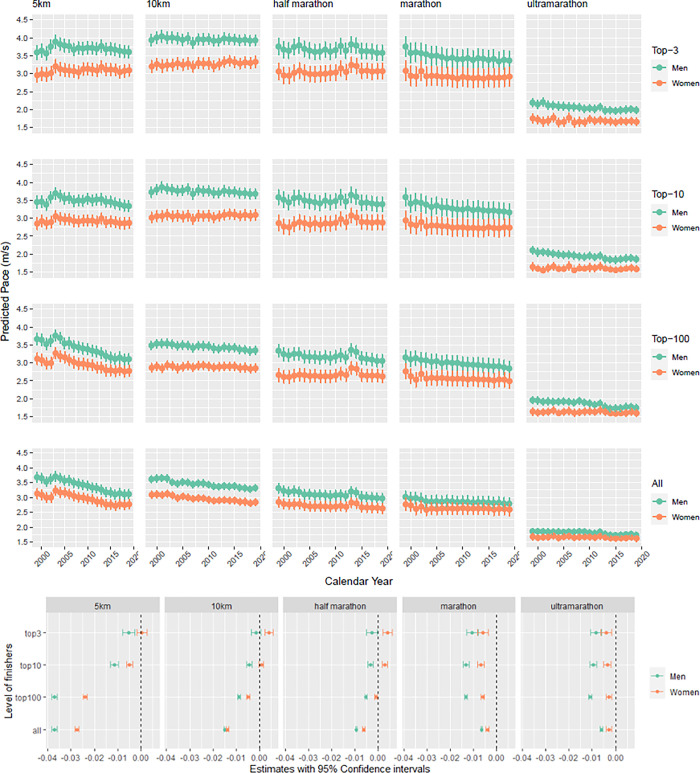
Predicted running speed. **A.** Predicted running speed (in m/s) by race distance, calendar year, sex and rank (Top 3, Top 10, Top 100, All) derived from the linear mixed models adjusted for age and competition. **B. Linear change in running speed.** Estimates for linear change in running speed (in m/s) per year by race distance, calendar year, sex and rank (Top 3, Top 10, Top 100, All) derived from the linear mixed models adjusted for age and competition.

Within all race distances, men were running faster than women. We observed a decrease in running speed over the years in men and women in all running distances (Fig 9A). The decrease in running speed was lower when considering only the top-3 or top-10 finishers of each race distance and speed even increased in women participating in 10 km and half marathon races (Fig 9B).

### Running speed by age, sex and distance

**Fig 10:**
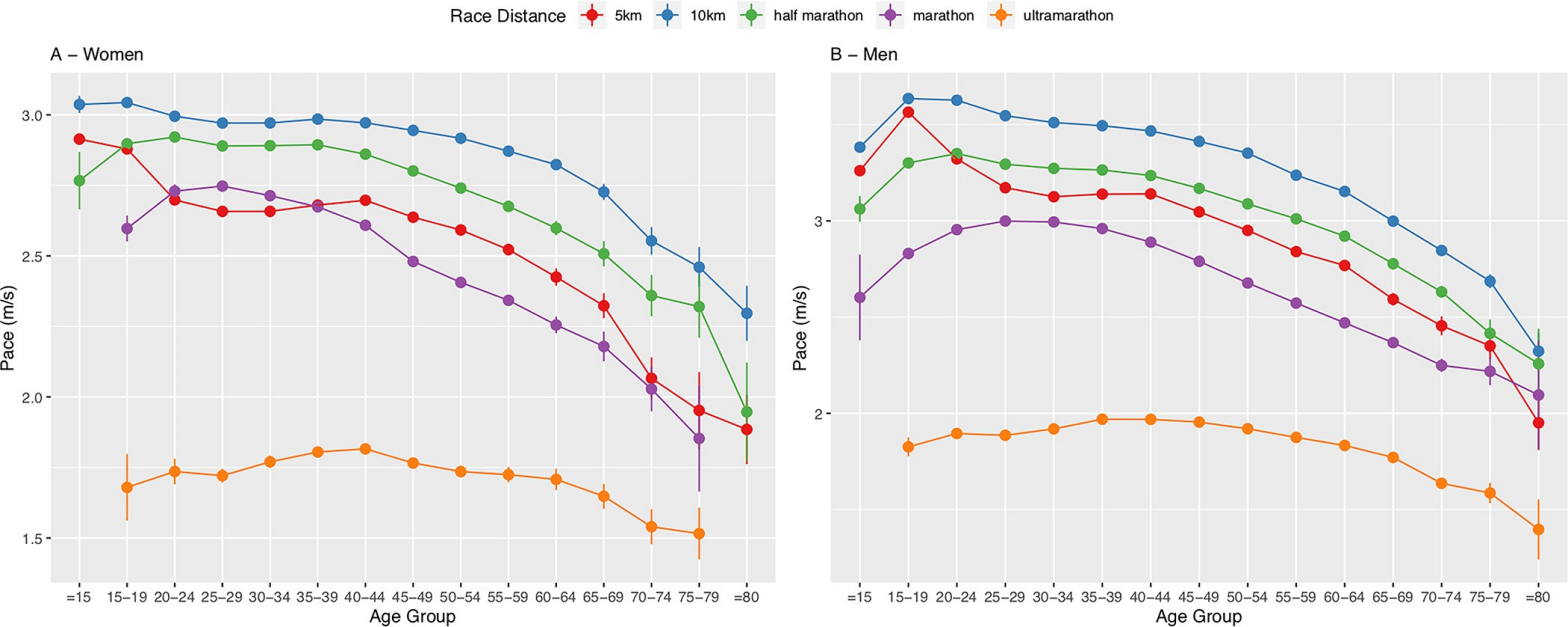
Running speed (in m/s) by age, sex and race distance.

In both men and women, older finishers were running slower than younger age group runners for all distances (Fig 10). Finishers in the 10 km races were the fastest and finishers in ultra-marathons were the slowest for both sexes and all age groups. Men were running faster in all age groups and distances. In ultra-marathons, the gap between men and women was the closest, whereas the largest gap between the sexes was in 10 km races. No women finished in marathon and in ultra-marathon in age group > 80 years.

## Discussion

This study intended to analyze the participation and performance trends of 1,172,836 age-grouped athletes competing in 5 km races, 10 km races, half-marathon, marathon, and ultra-marathon in Switzerland between 1999 and 2019. The most important findings were: (1) an increase in the number of finishers only in ultra-marathon in the most recent years, (2) a significant increase in finishers in trail run, (3) a low men-to-women-ratio in 5 km races, (4) both sexes were running slower in all race distances across the years, (5) men were running faster than women in all race distances, (6) a lower performance difference between men and women in longer race distances, (7) a longer running distance correlates with a higher age and age decreased in ultra-marathon in recent years, (8) the youngest ultra-marathoners were the fastest ultra-marathoners in recent years and (9) athletes in the 10 km races were the fastest compared to the other race distances.

The first important finding was the constant increase in the number of finishers in ultra-marathon in recent years, even if in total numbers, ultra-marathon races had the lowest number of finishers across years. In the other race distances (5 km, 10 km, half-marathon), there was an increase throughout the years, but not in recent years, whereas marathon–after an initial increase—lost constantly on participants. Data covering race results from the whole world (1986–2018) have shown similar results with a running participation that declined 13% from 2016 to 2018 [10].

Even if 5 km races are the most popular running distance in the USA [8], we do not see this trend in Switzerland, as 5 km races account for one of the lowest numbers of finishers. One reason could be that these are rather local events and attract less international athletes: for example only five Swiss 5 km races are listed on an international running platform, compared to 15 well-known Swiss marathons [42]. Concerning the 10 km race, the decrease in the number of finishers in the last years could be due to the modified length of some races (e.g. ‘Silvesterlauf Zürich’), leading to the exclusion from our study.

An exponential increase in the number of finishers in ultra-marathons was seen by several authors in the USA [43] and worldwide [25,27] and is mainly due to an increase in male runners [31]. Moreover, it follows the general trend to participate in ultra-endurance disciplines, such as ultra-distance swimming [44] or ultra-triathlon [45]. There is a growing interest in athletes to perform better, longer and under extreme conditions [46]. Thus, ultra-marathoners in Switzerland follow the worldwide trend of searching for new challenges beyond the marathon distance [24], which at the same time explains the decreasing numbers of participants in marathon.

The significant increase in the numbers of finishers in ultra-marathon is mainly due to the increase in ultra-trail runs, which represents our second important finding. At the same time, the number of finishers in road races decreased, maybe due to an increase in the offer of trail running events in Switzerland [41]. Switzerland, as being a mountainous and nature-orientated country, lends itself for long and diversified trail runs. Future studies are needed to analyze the participation trends in ultra-marathons after 2019, with regard to the Covid-19 pandemic [47].

A third important finding was that only in 5 km races, more women participated compared to men, which is in line with data from the USA [10]. Regarding 10 km races, our data differed from existing literature [9], as we found a higher MWR. This could be due to the number of analyzed 10 km races and the observed countries. In terms of long-distance races, the higher number of male finishers is in agreement with existing literature [9,27].

Women started later to participate in large running events [48], as they have been officially allowed only in 1972, and though first started running in shorter race distances, as it is reflected by the MWR of the 5 km and 10 km races. Compared to the USA, more female half-marathoners have been noted than male half-marathoners [16], which shows that the MWR might also vary from country to country. Further studies might evaluate the differences in sex and race distances in other countries and investigate if the longer race distances gain in female athletes in the following years.

A fourth important finding was that across years, men and women were running slower in all race distances, which is in accordance to several studies [1,15,49]. In 5 and 10 km races this could be explained by the simple increase in the numbers of finishers, as many newcomers and thus comparatively slower runners were entering the race [11]. Since the 1990s, a second boom has been observed in running with most of the participants simply “competing to complete” a race [50]. The short distance running events become more and more social events, possible to be performed by everybody.

Concerning half-marathon, Knechtle et al. found that only female half-marathoners were running slower, but performance in male half-marathoners remained unchanged across years, whereas performance in marathon did not change for both men and women across years [12]. One explanation for these different findings could be the different periods of time of the studies, as the main decrease in performance in our study was mainly seen after 2014 and Knechtle et al. only analyzed data before 2014.

For ultra-marathon, one potential explanation could be again the increasing number of trail runs in recent years, which involves longer running times. As many Swiss trail runs have big differences in altitude, like the ‘Eiger Ultra Trail’ with a height difference of 6,700 m, they demand more effort from the athletes than flat ultra-marathons. Future studies should investigate if this trend is also seen in other sports disciplines and if there would be again an upturn of the runners’ performances.

A fifth important finding was that men were running faster than women in all race distances. This finding is already described in literature for short races as the 5 km [10] and 10 km [9] as well as ultra-marathon races [17,29,49]. In marathon and half-marathon running, however, there are inconsistent findings in literature. Our data was in accordance to studies from Leyk et al. [20], Steffny [21] and Hunter [51], but differed from Knechtle et al., who found that women were running slightly, but still significantly faster than men [12]. A possible explanation is the different focus of the studies, as Leyk et al. and Hunter focused on the top-ten runners, whereas Knechtle et al. analyzed all recorded race times of female half-marathoners. Comparing our data to Knechtle et al., we have different results as in our study, we included both road and trail races, whereas Knechtle et al. only included flat half-marathons and marathons.

Reasons for a better performance in men compared to women could be associated with physiological differences or social and environmental aspects. Regarding the physiological characteristics, a body of evidence has highlighted differences such as a lower aerobic capacity and a lower muscular strength in women as well as differences in anthropometric (i.e., body composition), thermoregulatory and metabolic characteristics [52–54]. In addition, since running is an outdoor practice, the social and environmental characteristics can impair the female training commitment. Many barriers influence women in performing a physical outdoor activity, as for example personal safety, lack of self-confidence or lack of time and childcare [55]. Training habits also play a role as for example male half-marathoners had faster running speeds during training than women [56], more and longer weekly training units as well as more experience in sports than women [57].

Even if men run faster than women in all race distances, the performance difference between the sexes decreased by increasing race distance as our sixth important finding. The sex difference in performance was the highest in 5 km and 10 km races and the lowest in ultra-marathon and marathon races, which is in accordance to Sousa et al. [58]. This fact can be explained by a decreasing number of female finishers with increasing race distance [9] and thus probably a higher participation of women with running experience in longer compared to shorter races, which count many slower newcomers.

In ultra-marathon, the decreasing performance difference between men and women across years was in accordance with Knechtle et al. concerning most of the timed ultra-marathons [29] and with Hoffmann et al. concerning the 161-km (100 miles) ultra-marathons in North America [43]. In American road running events however, Sousa et al. found that the performance gap between men and women did not decrease with increasing length [58]. One reason for this could be that the study did not include trail runs, why it cannot be extrapolated to Switzerland, being a country with a high percentage of trail runs. Future studies might investigate if the lower performance difference between men and women in longer distances is also seen in other sports disciplines, such as cycling and swimming.

In terms of age, we found that a longer running distance correlates with a higher median age and vice versa, which represents our seventh finding. Within the different race distances, finishers in short races such as 5 km and 10 km had a lower median age than finishers in longer races such as marathon and ultra-marathon, which is in accordance to a study from Knechtle et al. concerning Swiss half-marathons and marathons [12]. Interestingly, looking at data from the USA, we find the opposite as marathoners were younger than half-marathoners [59]. Regarding these different results, it is important to mention that the data from the USA only represent a single year compared to the data from Knechtle et al. in recent years and our data, which included 15 vs. 20 years [12].

In shorter running distances as for example the 5 km races, we find rather younger finishers than older finishers, which is in accordance to data from the USA [10]. One reason could be that older athletes are rather trained for endurance events and not for short races, where a fast and short speed-up is needed. This can be due to the normal human muscular atrophy in aging runners, showing at the same time a decrease in fast type II fibers with a remaining level of slow fast I fibers [60]. Consequently, a decline in rapid force capacity [61] and a slowing of contractile speed in muscle cells are seen [60]. Moreover, short races are often amusement events like Christmas or City runs [62,63], which attract rather the younger population and newcomers.

Ultra-marathon running had the highest median age compared to all shorter distances. One reason could be that the athletes in longer distances already have more running experience [24,64], as they started running with shorter distances in younger years [65]. Nevertheless, median age in ultra-marathoners decreased constantly after 2012, approaching the median age of marathon races. This is an interesting finding, reflecting that younger athletes are more and more getting attracted to the rising offer of ultra-marathon events in general, and especially of trail runs, which show an important increase as indicated above. Athletes in Swiss ultra-marathons even have a lower median age than it is described in literature as for example 44.5 years in North America [66]. Future studies might investigate if this trend continues in the following years.

Regarding performance, the age of peak performance in 5 km races lies in age groups < 20 years for both sexes in our study, which is in accordance to raw data of the USA [10]. In 10 km races, it lies ≈ 20 years for both sexes, which is also in accordance to previous studies [9,67]. The best performance appears to decrease after 40–44 years. An explanation could be the natural aging process that affects the cardiovascular function (i.e. maximal heart rate, cardiac output and aerobic capacity), the decrease in arteriovenous oxygen differences [68] and the progressive loss of tissue function [69]. Given that performance in a 10 km distance race depends mostly from aerobic capacity [70] and running economy [71], performance decline across age is strongly influenced by these factors [72].

In all race distances, older age groups showed the lowest performance (i.e. a decrease in performance with an increase in median age), which was in agreement with previous studies [9,10,18,33,73]. Future studies might investigate the physiologic causes for the better performance in 10 km races compared to 5 km races.

An eighth important finding was that since 2016, the youngest ultra-marathoners (<35 years) were the fastest. Before 2016, the peak performance in both sexes lies in age group 35–55 years, which is in accordance to literature [24,73,74]. Finishers > 55 years were running slowest across years, having a lower endurance capacity due to the decrease in the cardiopulmonary system and a declining force generation by skeletal muscle [75]. This finding has an important medical impact, as the increase in excessive endurance-based activity in older athletes not only leads to long-term benefits on physical health such as preventing cardiovascular diseases and psychological wellbeing [5], but may also have adverse effects on the cardiovascular system. Some studies found that endurance-based activity may also cause reversible electrocardiographic changes and induce a pathologic structural remodeling of heart and large arteries, leading to cardiovascular diseases such as atrial fibrillation, coronary calcifications or myocardial fibrosis [76,77]. Those facts will challenge doctors to provide appropriate medical care with an aging but active population.

Our last important finding was that female and male finishers showed their best running times in 10 km races, but also showed a better average running speed in half-marathon than in 5 km races. One possible explanation could be that many novice runners participate in 5 km races, who tend to have slower running times, and that 5 km races often are amusement runs in which the sole participation is important and not the performance of each athlete. Concerning the 10 km race distance, it is often used as a training race distance regarding longer races such as half-marathons and marathons [9,78]. This is one possible reason why athletes perform best in this race distance.

Our current findings are relevant for race directors, who might need to follow the race trends. They should focus more on races like ultra-marathon instead of offering events of decreasing interest. The fact that older athletes more and more attend longer race distances has a big impact on their medical attendance, as they often have pre-existing illnesses and as endurance running increases the risk e.g. of atrial fibrillation or bone-joint diseases.

## Conclusions

In summary, for athletes competing from 1999 to 2019 in 5 and 10 km races, half-marathon, marathon, and ultra-marathon in Switzerland, we showed that they have a growing interest in ultra-marathon events, especially in trail runs, and that finishers were getting older across years with increasing race distance. In recent years, ultra-marathoners showed a downward tendency in the age of the finishers. Moreover, we demonstrated that athletes lost on performance across years within all race distances and that differences in the performance between men and women persist, even if they decrease by increasing race distance. Future studies should be performed concerning running trends in other countries. They might also investigate if the participation trends towards ultra-marathon races and the increasing age of the runners would continue in the next years, considering especially the years with the Covid-19-pandemic, and if there would be again an upturn of the runners’ performances.

## Supporting information

S1 Fig 5 km race events and number of races. Number of finishers in 5 km races divided into the different events (upper panel) and number of 5 km races over calendar years (lower panel).
(PDF)

S2 Fig 10 km race events and number of races. Number of finishers in 10 km races divided into the different events (upper panel) and number of 10 km races over calendar years (lower panel).
(PDF)

S3 Fig Half-marathon events and number of races Number of finishers in half-marathon races divided into the different events (upper panel) and number of half-marathon races over calendar years (lower panel). (PDF)

S4 Fig Marathon events and number of races Number of finishers in marathon races divided into the different events (upper panel) and number of marathon races over calendar years (lower panel). (PDF)

S5 Fig Ultra-marathon events and number of races Number of finishers in ultra-marathon races divided into the different events (upper panel) and number of ultra-marathon races over calendar years (lower panel). (PDF)

S6 Fig Running speed (in m/s) by age, sex and race distance. (PDF)

S1 File Whole dataset of 5km, 10km, half-marathon, marathon, ultra-marathon races from 1999–2019. (XLSX)
