# Supplementary figures and images for: Running trends in Switzerland from 1999 to 2019: An exploratory observational study
Source: PLoS One. 2025 Jan 16;20(1):e0311268. doi: 10.1371/journal.pone.0311268 (PMC11737753; doi:10.1371/journal.pone.0311268)

# 5km Races

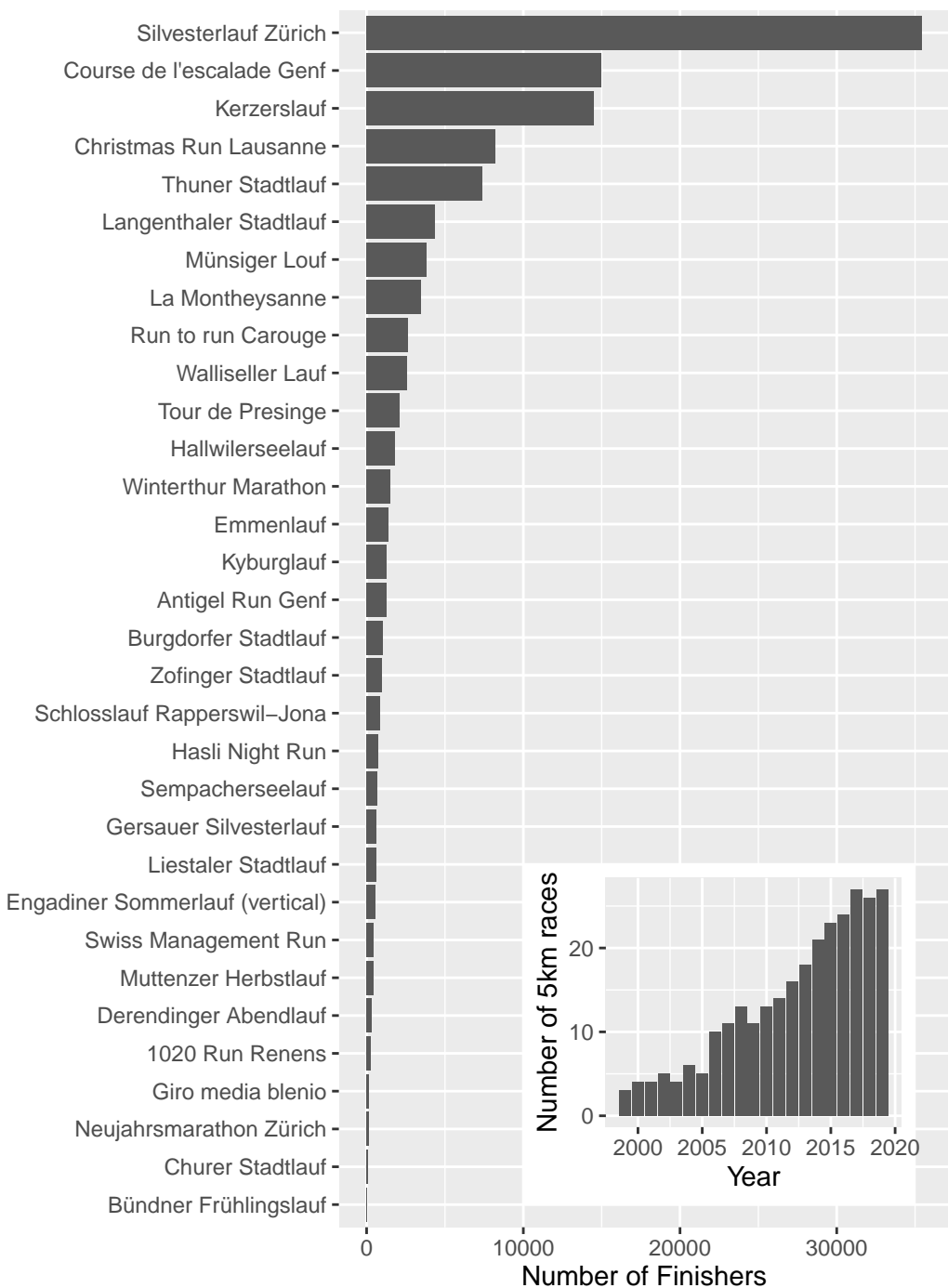

Supplement: S1 Fig — Number of finishers in 5 km races divided into the different events (upper panel) and number of 5 km races over calendar years (lower panel). (PDF) [file pone.0311268.s001.pdf]

# 10km Races

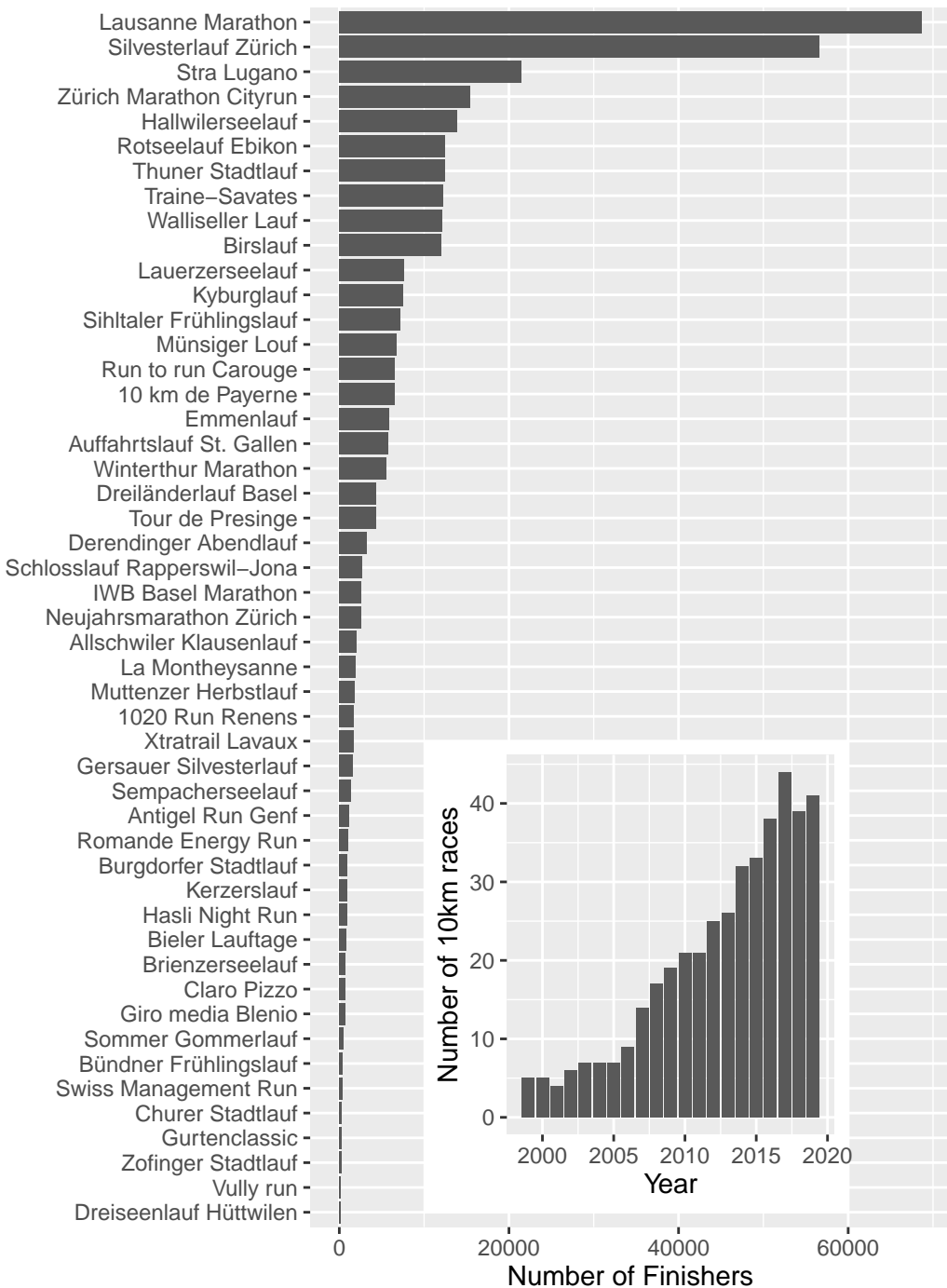

Supplement: S2 Fig — Number of finishers in 10 km races divided into the different events (upper panel) and number of 10 km races over calendar years (lower panel). (PDF) [file pone.0311268.s002.pdf]

# half marathon Races

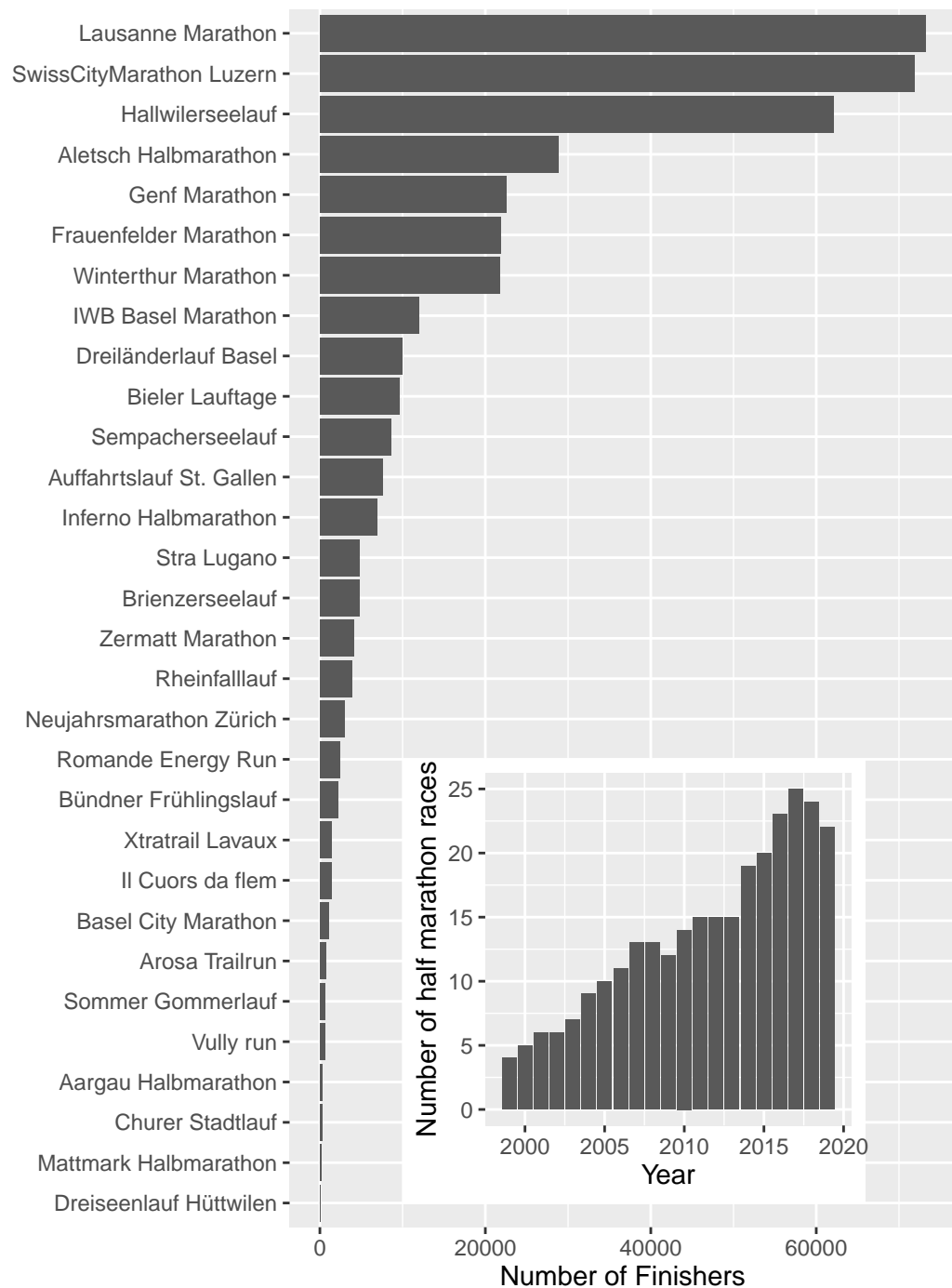

Supplement: S3 Fig — (PDF) [file pone.0311268.s003.pdf]

# marathon Races

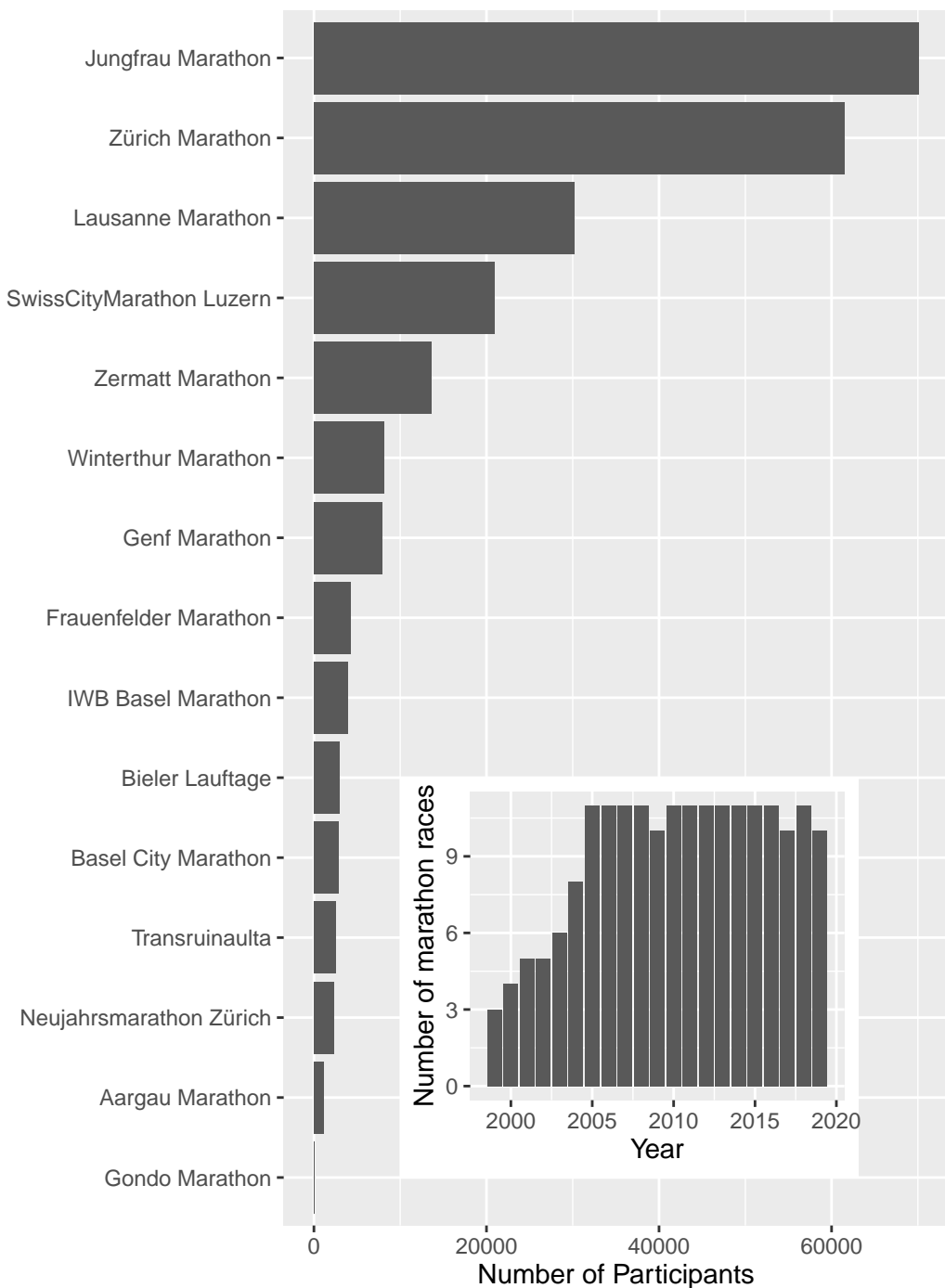

Supplement: S4 Fig — (PDF) [file pone.0311268.s004.pdf]

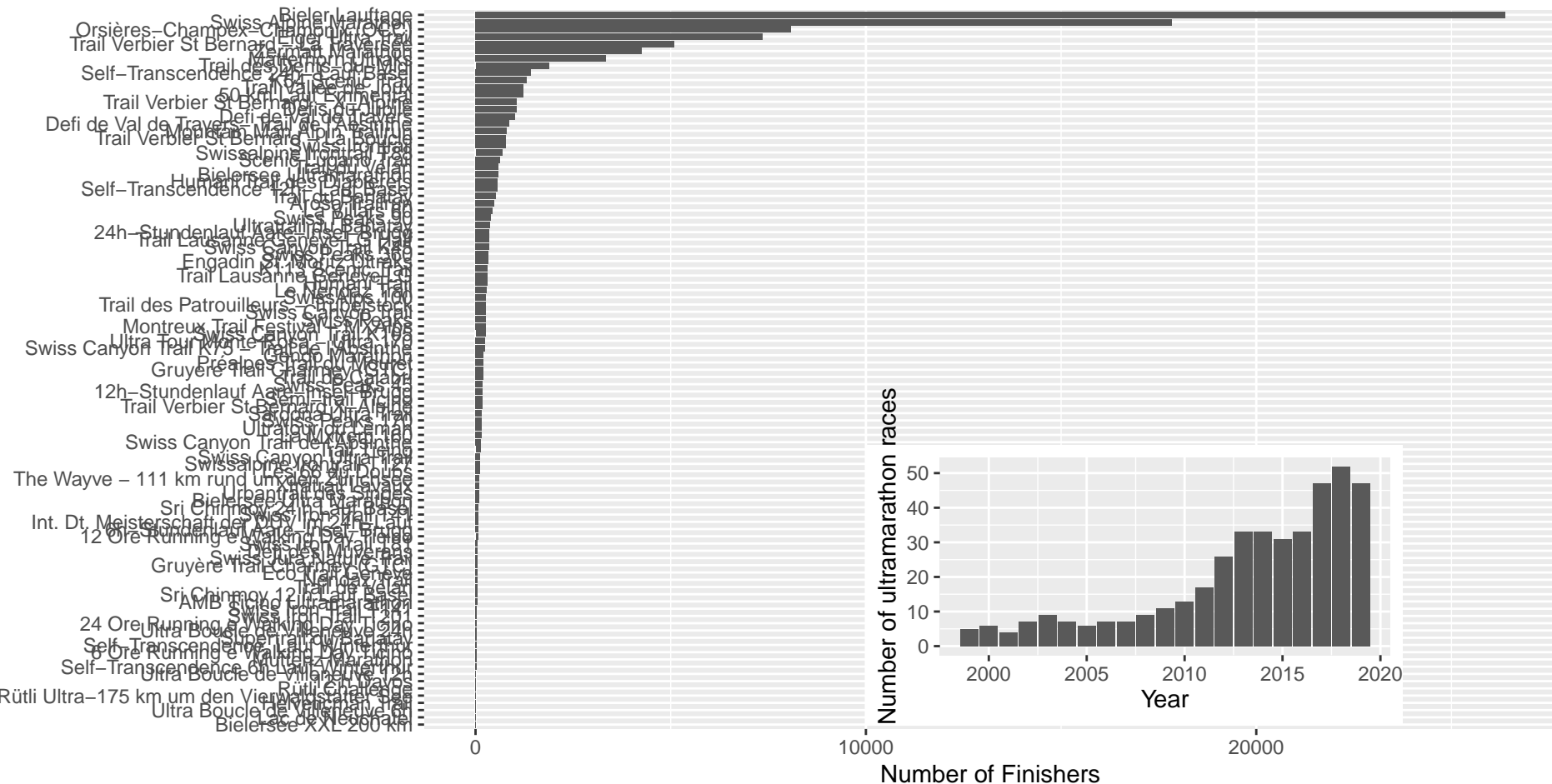

Supplement: S5 Fig — (PDF) [file pone.0311268.s005.pdf]

Race Distance    5km    10km    half marathon    marathon    ultramarathon

A – Women

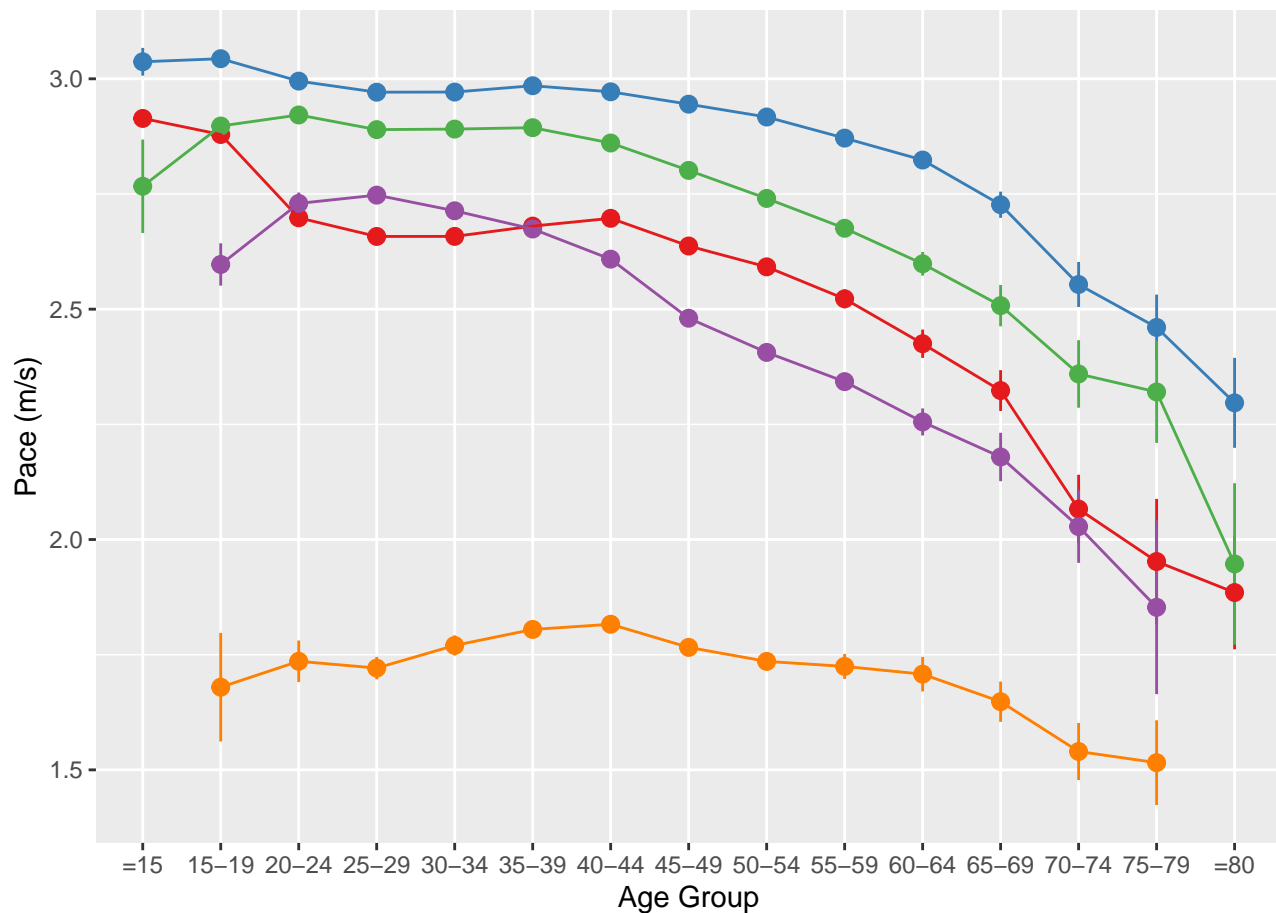

B – Men

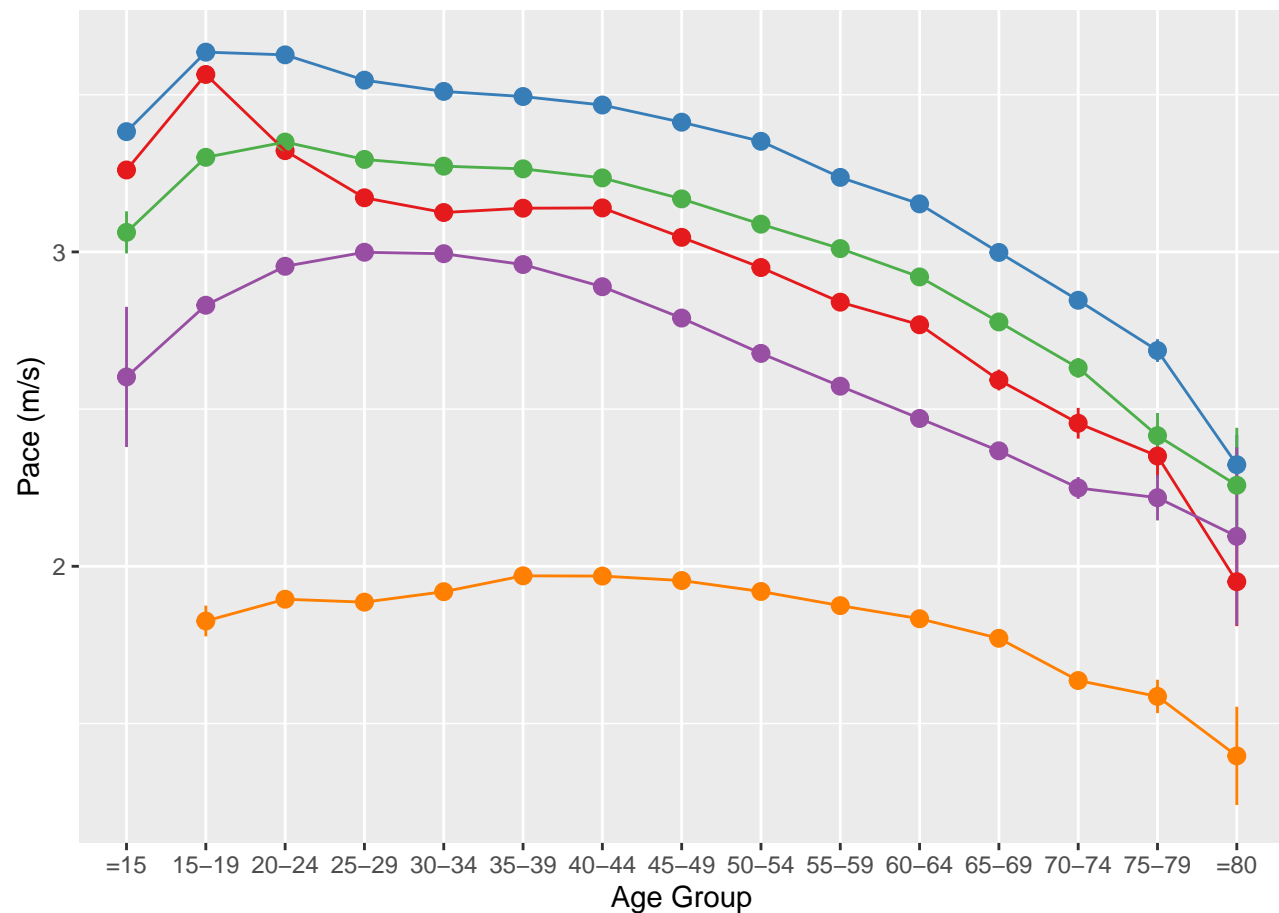

Supplement: S6 Fig — (PDF) [file pone.0311268.s006.pdf]
